# AGING AND DECISION MAKING: THE ROLES OF AFFECTIVE AND SOCIAL CONTEXT

**DOI:** 10.1093/geroni/igad104.0093

**Published:** 2023-12-21

**Authors:** Julia Nolte, Kendra Seaman

## Abstract

Age differences in many facets of decision making are well-established. However, a growing literature suggests that the occurrence and strength of these age effects are moderated by contextual factors. The individual talks in this symposium exemplify how age differences in decision making vary by domain, affective intensity, and social distance. First, Nolte and Löckenhoff demonstrate age-related increments in the avoidance of health-based and consumer choices, finding that perceived difficulty, but not affective responses to choice, increase avoidance. Frank and Pachur highlight that affective responses to choice differ across contexts: Younger and older adults exhibit “affect gaps” by making less beneficial, more risk-averse choices in “affect-rich” (health-based) versus “affect-poor” (consumer) contexts. Contrasting monetary and temporal outcomes, Strough, Bruine de Bruin, and Parker illustrate that the tendency to continue failing investments is more pronounced after investing money than time. Moreover, older adults’ decreased susceptibility to this “sunk cost bias” is more evident when making choices about their own investments versus others’ investments. Lu and Löckenhoff report age differences in how decision makers discount other’s financial well-being as social distance increases: Older adults’ willingness to share money with others declines more with increasing social distance than is the case for younger adults. Finally, Wild and Löckenhoff demonstrate that across domains, older versus younger decision makers are less willing to consult with distant social partners or experts and consult fewer people fewer times. To identify directions for future research, Seaman will contextualize the five presentations against the extant aging and decision-making literature.

